# Diagnostic value of magnetic resonance imaging for malignant ovarian tumors mis-subclassified by the ultrasound-based ADNEX model

**DOI:** 10.3389/fonc.2025.1406735

**Published:** 2025-02-25

**Authors:** Meijiao Jiang, Congcong Yuan, Siwei Lu, Yunkai Zhu, Caiting Chu, Wenhua Li

**Affiliations:** ^1^ Department of Radiology, Xinhua Hospital, Shanghai Jiao Tong University School of Medicine, Shanghai, China; ^2^ Department of Ultrasound, Ruijin Hospital, Shanghai Jiao Tong University School of Medicine, Shanghai, China; ^3^ Department of Ultrasound, Xinhua Hospital, Shanghai Jiao Tong University School of Medicine, Shanghai, China; ^4^ Department of Radiology, Xinhua Hospital, Shanghai Jiao Tong University School of Medicine, Chongming Branch, Shanghai, China

**Keywords:** malignant ovarian tumors, magnetic resonance imaging, diagnosis, ADNEX model, ultrasound

## Abstract

**Objective:**

Accurately predicting metastatic cancer to the adnexa, stage I and advanced ovarian cancer before surgery is crucial. The ADNEX model, based on ultrasound, is currently the only prediction model that can differentiate between these types. This study aims to analyze MRI features and diagnostic value in malignant ovarian tumors mis-subclassified by the ADNEX model, considering their diverse histopathologic types.

**Methods:**

From January 2018 to September 2022, 164 patients with pathologically confirmed ovarian malignancies were selected from those who were examined by ultrasound. The clinical and MRI characteristics of 51 patients mis-subclassified by the ADNEX model were compared with histopathological types.

**Results:**

A total of 30 were confirmed with primary ovarian cancer (5 with HGSOC, 14 with CCC, 2 with EC, 4 with MC, 2 with GCT, 1 with YST, 1 with immature teratoma, and 1 with dysgerminoma). There were 21 patients who had metastatic ovarian tumors (10 with colorectal cancer, 4 with gastric cancer, 2 with uterine cervical cancer, 3 with endometrial cancer, 1 with breast cancer, and 1 with LAMN). The only significant difference between the two groups was in CEA. The mean diameters of the primary and metastatic ovarian tumors were 10.29 cm (range: 3.61 cm–26.02 cm) and 8.58 cm (range: 3.10 cm–20.30 cm), respectively. A total of 42 masses were lobulated (82.35%, 42/51), and 26 masses were solid-cystic (26/51, 50.98%). There was a significant difference between CCC and other tumors, with mean ADC values of 1.01 × 10^−3^ mm^2^/s (range: 0.68–1.28×10^−3^ mm^2^/s) and 0.74×10^−3^ mm^2^/s (range: 0.48–0.99×10^−3^ mm^2^/s), respectively (P=0.000). A total of 50 masses presented isointense-T1, hyperintense-T2, and hyperintense-DWI signal on MRI (50/51,98.04%). There were 33 masses that showed intensive enhancement (33/51,64.71%). There were 17 masses who had necrosis (17/51, 33.33%), with the majority being HGSOC and ovarian metastases from colorectal and gastric cancers (12/17, 70.59%). There were 19 masses that presented hemorrhage (19/51,37.25%), with the majority being CCC (10/19, 52.63%). A total of 46 masses were diagnosed correctly by MRI (46/51,90.20%). There were 35 and 15 masses that were rated as O-RADS score 5 and score 4, respectively. One mass was rated as score 3.

**Conclusions:**

DWI signal, ADC value, degree of enhancement, and characteristic components within the mass on MRI can provide supplementary information for malignant ovarian tumors mis-subclassified by the ADNEX model.

## Introduction

As the most lethal gynecological malignancy, ovarian cancer represents the fifth leading cause of cancer-related deaths in women (1). According to statistics, the 5-year survival rate is less than 30% in patients with advanced stage ovarian cancer, which, by contrast, is more than 90% in patients of stage I ovarian cancer (2). Simultaneously, distinct treatment strategies are needed for primary and metastatic ovarian tumors. Therefore, accurately predicting the stage and origin of malignant ovarian tumors holds great significance in determining the most appropriate management strategy to prolong life, whether through surgical intervention or chemotherapy.

Preoperative imaging assessments, including ultrasound and magnetic resonance imaging (MRI), are crucial for evaluating patients with suspected ovarian tumors. Over the past two decades, various predictive models and scoring systems have been developed based on these imaging techniques (3–10), with a focus on predicting the risk of malignancy. Based on the consensus statement on preoperative diagnosis of ovarian tumors (11), the utilization of ultrasound assessment conducted by an expert or the application of the International Ovarian Tumor Analysis (IOTA) Assessment of Different NEoplasias in the adneXa (ADNEX) model (12) frequently enables the identification of the specific subtype of malignancy [borderline ovarian tumor (BOT), stage I ovarian cancer (stage I OC), stage II–IV ovarian cancer (stage II–V OC), and metastatic cancer to the adnexa]. Several previous studies have demonstrated the excellent performance of the ADNEX model in distinguishing between benign and malignant masses (13–16). In addition, the IOTA ultrasound-based ADNEX model has been found to exhibit comparable sensitivity to MRI in differentiating adnexal tumors, while also displaying higher specificity and accuracy in identifying borderline tumors (17). Nevertheless, there is a need for further enhancement in the accuracy rate of this model specifically in the prediction of subtypes of malignant ovarian tumors (13–16). In clinical practice, MRI, particularly contrast-enhanced MRI and diffusion-weighted Imaging (DWI), is generally considered as the next evaluation step when some ovarian masses could not be determined by ultrasonography (11, 18). Published studies usually focus on MRI’s diagnostic value for sonographically indeterminate ovarian masses (19, 20). To our knowledge, there has not yet been a study that examines the clinical and MRI features of ovarian mass cases where the ADNEX model has inaccurately predicted outcomes.

Consequently, this study aims to analyze the histopathological types, and the clinical and MRI characteristics of malignant ovarian tumors mis-subclassified by the ADNEX model. This effort may pave the way to the improvement of this model or development new prediction models based on MRI, in order to refine the preoperative diagnostic accuracy for patients with malignant ovarian tumors.

## Materials and methods

### Study population

Participants presenting with suspected ovarian tumors on ultrasound in our hospital were enrolled between January 2018 and September 2022. Subsequently, a total of 164 patients with pathologically confirmed ovarian malignancies, encompassing both primary and metastatic tumors, were selected for the study. All of the imaging data were stored in the picture archiving and communication system (PACS). The interval between operation and examination (ultrasound and MRI) of these patients did not exceed 120 days. Moreover, the patients had no previous history of ovarian cancer. The exclusion criteria were as follows: 1) the ovarian masses were subclassified correctly by the ADNEX model; 2) no enhanced MRI was performed before surgery.

Clinical information, including Cancer Antigen 125 (CA125), Cancer Antigen 199(CA199), Carcinoembryonic Antigen (CEA), and human epididymis protein 4 (HE-4) test results, with different histopathologic types, were analyzed if detected. In this study, patients whose ovarian tumors was mis-subclassified by the ADNEX model were categorized into two groups: primary ovarian cancer (including both I and II–IV stages) and metastatic ovarian tumor. Their age, menopausal status, and relevant tumor markers were presented separately, and above data between the two groups were compared. All assessments were performed prior to surgery and chemotherapy.

### Ultrasound examination and the ADNEX model

All 164 patients presented with at least one adnexal mass and subsequently underwent transvaginal and transabdominal ultrasonography. The imaging was conducted using EPIQ5 ultrasound machines (Philips Health Systems, Bothell, WA, USA) and Vivid E95 ultrasound machines (GE Healthcare), equipped with a 7.0 MHz–9.0 MHz transvaginal probe and a 3.5-MHz transabdominal probe.

Experienced ultrasonographers preoperatively assessed sonographic tumor morphology according to the IOTA consensus about the terms, definitions, and measurements used to describe the ultrasound features of adnexal tumors in 2000 (21). Multiangle scans were performed to obtain more information about the masses from ultrasound images. For bilateral ovarian masses, the mass with the most complex ultrasound features was included to the ADNEX model. If two masses had similar ultrasound morphologies, the largest mass or the one most easily accessible by ultrasonography was included (13, 21).

We input the variables needed into the ADNEX model paid for from the Apple store. The model includes nine variables: age (years), serum CA125 level (U/mL), type of center (oncology referral center vs. non-oncology center), maximal diameter of the lesion (mm), maximal diameter of the largest solid part (mm), number of papillary projections (0, 1, 2, 3, or more than 3), number of cysts locules (≤10 vs. >10), acoustic shadows (yes or no), and ascites (yes or no) (12). All ADNEX model parameters were logged objectively. In the final diagnosis, the masses were divided into five types: benign ovarian tumors, BOTs, stage I OC, stage II–IV OC, and metastatic cancer to the adnexa.

### MRI examination

MRI was performed using a 3.0 Tesla (T) MR superconductor unit (TwinSpeed, GE Medical Systems, Milwaukee, WI, USA). A pelvic phased-array coil was employed for all cases. The following unenhanced sequences were acquired: axial T1-weighted imaging (T1WI) with a time of repetition (TR) of 340 ms and a time of echo (TE) of 10 ms; axial fast spin echo (FSE) T2-weighted imaging (T2WI) with fat saturation, using a TR/TE of 8,000 ms/83 ms; sagittal FSE T2WI with a TR/TE of 8,000 ms/98 ms. An axial DWI was performed with a b value of 1,000 s/mm^2^, utilizing a TR/TE of 3350 ms/67.6 ms. Apparent diffusion coefficient (ADC) maps were automatically generated. Contrast-enhanced T1WI LAVA 2D with fat saturation was conducted in the axial, sagittal, and coronal planes following the injection of gadopentetate dimeglumine (Gd-DTPA, 0.1 mmol/kg of body weight, Magnevist; Bayer Schering, Guangzhou, China) at a rate of 2 mL–3 mL.

### MR Image analysis and O-RADS MRI score

MRI features of the tumors were assessed including the following. 1) Tumor shape and tumor size. Maximum diameter of the mass was measured. 2) Tumor T1WI, T2WI, and DWI signal intensity on MR image. The masses were rated as hypointense (lower signal than outer myometrium), isointense (similar signal to outer myometrium), or hyperintense (higher signal than outer myometrium). 3) ADC value. On ADC maps, a circular region of interest (ROI) of at least 1 cm^2^ was placed at targeted areas in the solid components of tumor, by referring to conventional MR images. 4) Patterns of enhancement. The enhancement degree was rated as slight (weaker than muscles), moderate (between muscles and outer myometrium), or intensive (more obvious than outer myometrium). 5) Presence of necrosis or hemorrhage. 6) Composition of the mass. The composition of the mass was rated as solid (mass consists of at least 80% solid tissue), solid-cystic (solid tissue of mass was rated as larger solid portion), and multilocular-cystic (including mass without solid tissue, and solid tissue of mass was rated as papillary projection, mural nodule, irregular septation, and irregular wall). The composition of the mass was according to the O-RADS™ MR Lexicon Categories, Terms and Definitions (22), **Table 1** for details. 7) The diagnostic accuracy based on MRI. The MR images of 51 patients were rated by two radiologists.

**Table 1:** The O-RADS™ MR Lexicon categories, terms, and definitions involved in the composition of the mass (22).

Sub-term of solid tissue (must enhance and conform to one of these morphologies)	Definitions
Papillary projection	Enhancing solid component arising from the inner/outer wall or septation of an adnexal lesion, with a branching architecture.
Mural nodule	Enhancing solid component, measuring >3 mm, arising from the wall or septation of an adnexal lesion, with nodular appearance.
Irregular septation	Enhancing linear strand that runs from one internal surface of the cyst to the contralateral side demonstrating an uneven margin.
Irregular wall	Enhancing cyst wall demonstrating an uneven margin.
Larger solid portion	Enhancing component of an adnexal lesion that does not fit into the categories of papillary projection, mural nodule, or irregular septation/wall.

Radiologists used the Ovarian-Adnexal Reporting and Data System (O-RADS) MRI Risk Stratification System (4) to reassess misclassified malignant ovarian tumors by the ADNEX model. O-RADS MRI 1: No detectable pelvic mass. O-RADS MRI 2: Purely cystic mass, purely endometriotic mass, purely fatty mass, or absence of wall enhancement. O-RADS MRI 3: Absence of solid tissue. O-RADS MRI 4: Lesion with solid tissue enhancing ≤myometrium at 30 s–40 s on non-DCE MRI, with lipid content. Large-volume solid tissue enhancing lesion. O-RADS MRI 5: Lesion with solid tissue enhancing >myometrium at 30 s–40 s on non-DCE MRI, peritoneal, mesenteric, or omental nodularity or irregular thickening with or without ascites.

### Histopathology

The histopathological diagnosis of the tumors after surgical removal by laparoscopy or laparotomy was used as a reference standard. Tumors were staged according to the World Health Organization (WHO) classification of tumors (23), and malignant tumors were staged using the International Federation of Obstetrics and Gynecology (FIGO) standards (24).

### Statistical analysis

Statistical analysis was performed using SPSS version 22.0 (IBM Corp, Los Angeles, CA, USA) and MedCalc version 15.2.2 (MedCalc Software, Mariakerke, Belgium) software. The MRI characteristics of the tumors, patients’ clinical features and tumor marker levels were compared using the chi-square test for categorical data and the Mann–Whitney U-test for continuous data. Statistical significance was assumed at P < 0.05 for all comparisons. The kappa coefficients were calculated to assess the interobserver agreement between the two radiologists for imaging parameter analysis. Kappa values of 0.00–0.20, 0.21–0.40, 0.41–0.60, 0.61–0.80, and 0.81–1.00 indicated slight, fair, moderate, substantial, and almost perfect agreement, respectively.

## Results

**Figure 1:**
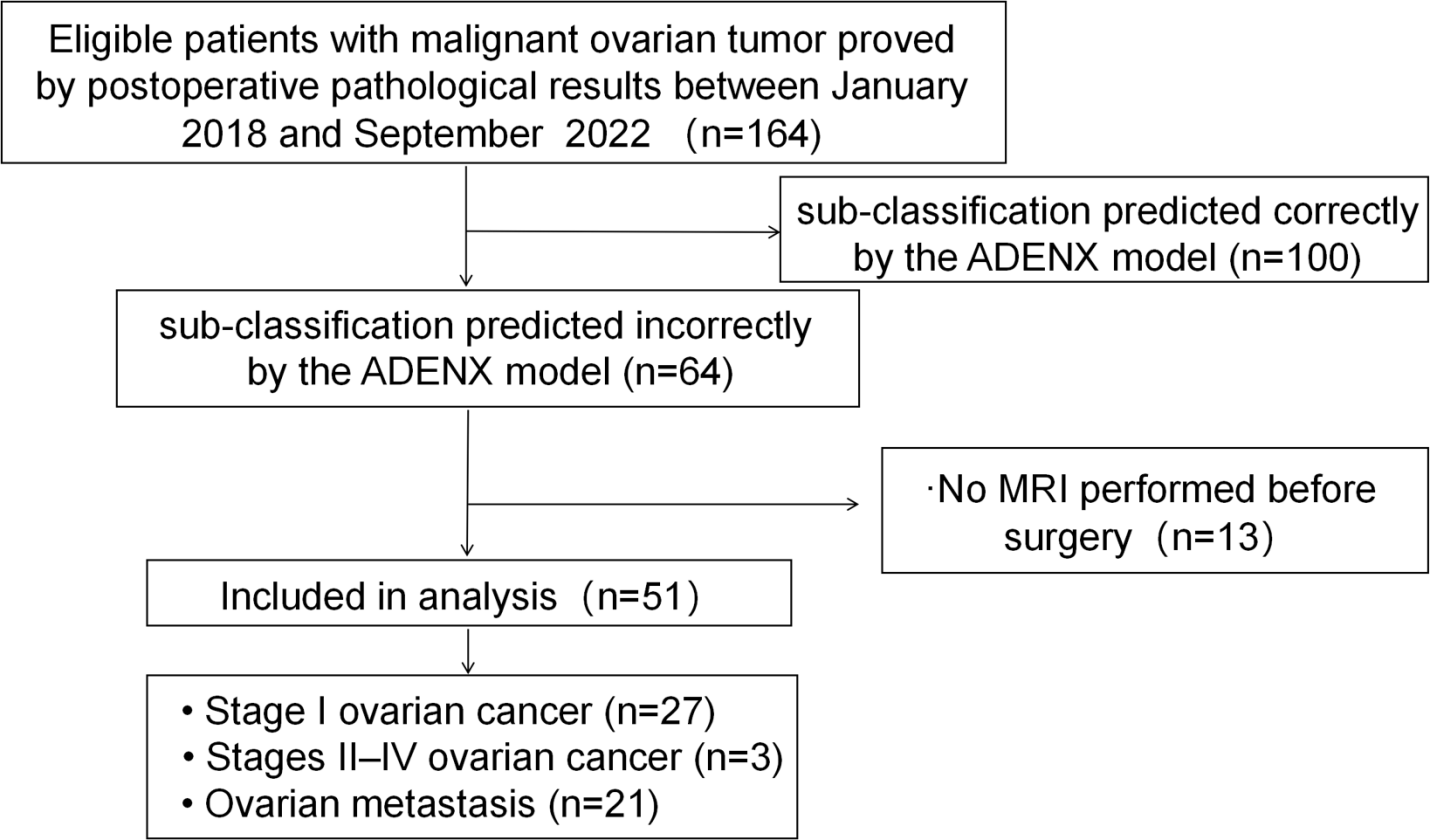
Flowchart showing the enrolment of women with malignant ovarian mass and reasons for exclusion in this study.

Between January 2018 and September 2022, 164 patients who underwent ultrasound examinations meeting the criteria received postoperative pathological assessments confirming malignant ovarian tumors. A total of 113 women were excluded from the study because the ADNEX model correctly subclassified their ovarian masses (n=100), and no contrast-enhanced MRI was conducted prior to surgery (n=13). Consequently, the final cohort comprised 51 patients (**Figure 1**).

### The results of subclassification errors of the ADNEX model

Among the 51 patients, 27 were confirmed to suffer stage I primary ovarian cancer. Within this group, the ADNEX model misdiagnosed four cases as benign tumors, 10 as BOTs, 12 as stage II–IV OC, and 1 as metastatic tumor. Additionally, three cases were confirmed as stage II–IV OC, with one case misdiagnosed as benign tumor and two as BOTs by the ADNEX model. Furthermore, 21 cases were confirmed as metastatic ovarian tumors, among which 8 were misdiagnosed as benign tumors, 6 as BOT, 1 as stage I OC, and 6 as stage II–IV OC.

### Histopathological results

Among the 51 patients, 30 were diagnosed with primary ovarian cancer, including 5 with high-grade serous ovarian carcinoma (HGSOC), 14 with clear cell carcinoma (CCC), 2 with endometrioid carcinoma (EC), 4 with mucinous carcinoma (MC), 2 with granulosa cell tumor (GCT), 1 with yolk sac tumor (YST), 1 with immature teratoma, and 1 with dysgerminoma. Additionally, 21 patients had metastatic ovarian tumors, with 10 having colorectal cancer, 4 having gastric cancer, 2 having uterine cervical cancer, 3 having endometrial cancer, 1 having breast cancer, and 1 having a low-grade appendiceal mucinous neoplasm (LAMN). **Table 2** shows the histopathological types of ovarian malignant tumors misclassified as benign or borderline tumors by the ADNEX model, whereas **Table 3** displays histopathological types of stage I ovarian cancers misclassified as more advanced stages and metastatic tumors.

**Table 2:** The histopathology types of ovarian malignant tumors which were mis-subclassified as benign and BOT by the ADNEX model.

	Stage I OC judged as benign by the ADNEX model (n=4)	Stage I OC judged as BOT by the ADNEX model (n=10)	Stage II–IV OC judged as benign by the ADNEX model (n=1)	Stage II–IV OC judged as BOT by the ADNEX model (n=2)	Metastatic cancer to the adnexa judged as benign by the ADNEX model (n=8)	Metastatic cancer to the adnexa judged as BOT by the ADNEX model (n=6)
Primary ovarian tumors(n=26)						
HGSOC (n=5)	1	NA	NA	NA	NA	NA
CCC (n=14)	1	7	1	1	NA	NA
MC (n=4)	NA	2	NA	1	NA	NA
GCT (n=2)	2	NA	NA	NA	NA	NA
Immature teratoma(n=1)	NA	1	NA	NA	NA	NA
Metastatic ovarian tumors(n=21)						
Colorectal cancer (n=10)	NA	NA	NA	NA	4	4
Gastric cancer (n=4)	NA	NA	NA	NA	1	NA
Uterine cervical cancer (n=2)	NA	NA	NA	NA	1	NA
Breast cancer (n=1)	NA	NA	NA	NA	NA	1
Endometrial cancer (n=3)	NA	NA	NA	NA	1	1
LAMN (n=1)	NA	NA	NA	NA	1	NA

BOT, borderline ovarian tumor; OC, ovarian cancer; HGSOC, high-grade serous ovarian carcinoma; CCC, clear cell carcinoma; MC, mucinous carcinoma; GCT, granulosa cell tumor; LAMN, low-grade appendiceal mucinous neoplasm.

**Table 3:** The histopathology types of stage I cancers that were mis-subclassified as stage II–IV and metastatic cancers by the ADNEX model.

	Stage I OC judged as stage II–IV OC by the ADNEX model (n=12)	Stage I OC judged as metastatic cancer to the adnexa by the ADNEX model (n=1)
HGSOC (n=5)	4	NA
CCC (n=14)	4	NA
EC (n=2)	2	NA
MC (n=4)	1	NA
YST (n=1)	1	NA
Dysgerminoma (n=1)	NA	1

OC, ovarian cancer; CCC, clear cell carcinoma; EC, endometrioid carcinoma; MC, mucinous carcinoma; YST, yolk sac tumor; HGSOC, high-grade serous ovarian carcinoma.

Furthermore, we summarized the pathological classifications of 100 cases that were accurately diagnosed by the model. Among the 100 patients, 91 were diagnosed with primary ovarian cancer, including 83 with HGSOC, 3 with CCC, 3 with EC, 1 with low-grade serous ovarian carcinoma (LGSOC), and 1 with small cell carcinoma of the ovary-pulmonary type (SCCOPT). Additionally, 9 patients had metastatic ovarian tumors, with 4 originating from colorectal cancer, 3 from gastric cancer, 1 from endometrial cancer, and 1 from high-grade appendiceal adenocarcinoma.

### Clinical information

All the patients’ relevant clinical indicators are presented in **Table 4**. Among the 51 patients mis-subclassified by the ADNEX model, no significant differences were found between the primary and metastatic groups regarding the aforementioned data, except for CEA (P=0.013).

**Table 4:** Clinical features of 51 patients who mis-subclassified by the ADNEX model.

	Primary ovarian tumors (n=30)	Metastatic ovarian tumors (n=21)	P value
Age	56.00(44.50-64.50)	51.50(41.75-64.00)	0.536
Postmenopausal status	19(0.63)	11(0.52)	0.265
CA125 (U/mL)	45.09(21.05-129.35)	34.00(16.20-173.50)	0.821
CA199 (U/mL)	11.78(6.14-22.05)	33.60(7.08-96.20)	0.031
CEA (ng/mL)	1.64(1.15-3.83)	7.34(1.57-18.55)	0.013
HE-4	81.30(43.01-235.00)	74.05(56.23-128.38)	0.947

CA125, cancer antigen 125; CA199, cancer antigen 199; CEA, carcinoembryonic antigen; HE-4, human epididymis protein 4.

### Interobserver agreement

For all MR imaging variables, the interobserver agreement was good (kappa=0.87–0.93; **Table 5**).

**Table 5:** Interobserver agreement of MR imaging variables.

MRI variables	Kappa
Shape	0.90
Composition of the mass	0.92
T1 signal	0.91
T2 signal	0.92
DWI signal	0.93
Enhancement degrees	0.88
Necrosis	0.87
Hemorrhage	0.91
O-RADS score	0.90

MRI, magnetic resonance imaging; DWI, diffusion-weighted Imaging; ADC, apparent diffusion coefficient; O-RADS, Ovarian-Adnexal Reporting and Data System.

### MRI findings

MRI findings of 51 malignant ovarian tumors mis-subclassified by the ADNEX model are presented in **Supplementary Table 1**. The mean diameter of the primary malignant ovarian tumors was 10.29 cm (range: 3.61 cm–26.02 cm), and the metastatic ovarian tumors was 8.58 cm (range: 3.10 cm–20.30 cm). In this study, 42 masses were lobulated (82.35%,42/51), A total of 26 masses were solid-cystic (26/51, 50.98%), with 18 solid masses and 7 multilocular-cystic masses. There was a significant difference between ovarian CCC and other histopathologic subtype tumors with mean ADC values of 1.01×10^−3^ mm^2^/s (range: 0.68×10^−3^ mm^2^/s-1.28×10^−3^ mm^2^/s) and 0.74×10^−3^ mm^2^/s (range: 0.48×10^−3^ mm^2^/s-0.99×10^−3^ mm^2^/s), respectively (P=0.000). A total of 50 masses presented isointense T1, hyperintense T2, and hyperintense DWI signal intensity on MR image (50/51, 98.04%), 33 masses were of intensive enhancement degree (33/51, 64.71%), 16 were moderate, and 2 were of slight enhancement degree. There were 17 masses that had necrosis (17/51, 33.33%), with the majority being HGSOC, ovarian metastases from colorectal and gastric cancers (12/17, 70.59%). There were 19 masses that presented hemorrhage (19/51, 37.25%), with the majority being ovarian CCC (10/19, 52.63%). A total of 46 masses were diagnosed correctly by the radiologists, and the subject diagnostic accuracy was 90.20%. The five cases of MRI diagnostic errors included one case of stage I HGSOC, one case of stage I CCC, and three cases of metastatic ovarian tumors (colorectal cancer, uterine cervical cancer, and LAMN). A total of 35 masses were rated as O-RADS score 5, 15 masses were rated as O-RADS score 4, and 1 mass was rated as O-RADS score 3.

## Discussion

As revealed in the present study, the ADNEX model exhibited, to some extent, subclassification errors in the following classifications: 1) ovarian cancer or metastatic ovarian tumors versus benign tumors or BOTs; 2) stage I OC versus stage II–IV OC, and 3) primary versus metastatic ovarian tumors. MRI visualizes malignant tumors by hyperintense DWI signal intensity and low ADC values. In addition, MRI holds the advantage of assessing adjacent organs more comprehensively and observing the distinctive components inside ovarian masses. Most of the 51 malignant ovarian masses mis-subclassified by the IOTA ADNEX model were lobulated, solid, or solid-cystic, intensive enhancement masses that presented iso-intense T1, hyperintense T2, and hyperintense DWI signal intensity on MR image. Furthermore, the clinical and MRI characteristics vary between different histopathologic types.

### The malignant ovarian tumors: mis-subclassified by the ADNEX model as benign or borderline tumors

There were more than half masses mis-subclassified as benign or borderline among the 51 cases (30/51,58.82%) by the ADNEX model. The primary factor contributing to these errors can be attributed to an insufficient evaluation of the solid components. However, in MRI, most of masses mis-subclassified as benign or borderline by the ADNEX model have been accurately diagnosed as malignant ovarian tumor (25/30, 83.33%). Most of the 30 masses presented solid and solid-cystic masses with hyperintense DWI signal and intensive enhancement of solid portion on the MR image. A majority of these malignant masses displayed obvious low ADC values (22/30, 73.33%), consistent with previous studies (25, 26). The O-RADS scoring system, based on MRI, can be instrumental in distinguishing malignant ovarian tumors from benign and borderline ovarian tumors. Among the 30 cases in this study group, a significant majority (21/30, 70.00%) received an O-RADS score of 5, suggesting a malignancy risk between 50% and 90%.

**Figure 2:**
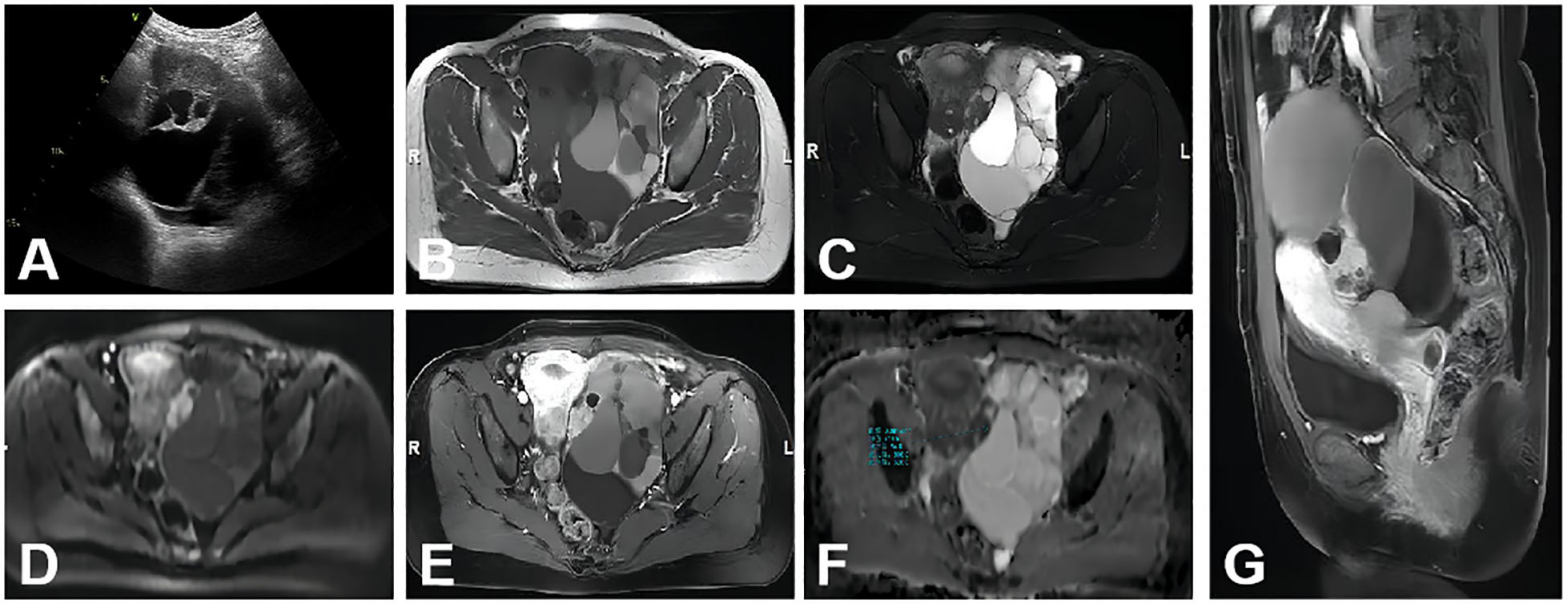
A 43-year-old woman with stage I MC on the left ovary. **(A)** A multilocular and predominantly cystic mass with thickened septa was detected by ultrasound examination. **(B–D)** A lobulated multi-cystic mass with mural nodule and irregular septations; the mural nodules showed mainly isointense, hyperintense, and hyperintense signal intensities on axial T1WI, T2WI, and DWI (b=1,000 s/mm^2^), respectively. **(E)** Axial contrast-enhanced T1WI showed that the mural nodule and the irregular septation were of moderate enhancement. **(F)** On the ADC map, the mean ADC value was 0.719 × 10^−3^ mm^2^/s. **(G)** Sagittal contrast-enhanced T1WI showed the mass was similar to **(E)**.

When the solid component is too small, it is more likely for the ADNEX model to predict the mass as a benign or borderline tumor. MRI has a higher capability to identify the solid constituents of masses that were not detected by ultrasound, particularly in cases when some special pathological types of ovarian tumors always present multi-cystic mass without apparent large solid portion such as mucinous neoplasms of the ovary (27) (**Figure 2**).

### Stage I OC: mis-subclassified by the ADNEX model as stage II–IV OC

From 12 cases in this group, we found that over half of these masses were type I epithelial ovarian cancers (EOC), consisting of four CCC, two EC, and one MC (7/12,58.33%). Concurrently, our study revealed that the ADNEX model demonstrated an accuracy of 0.94 (83/88) for HGSOC (the most common types of type II EOC), whereas its accuracy for CCC was markedly lower at 0.17 (3/17).

In comparison with type II EOC, type I EOC tended to exhibit a relatively indolent clinical course (28). Type I EOCs are usually detected in their early stages. In our research, type I EOC showed lobulated, large, solid-cystic masses with a large proportion of solid components, which was one of the variables input into the ADNEX model, potentially leading to the sub-classifications of the ADNEX model as stage II–IV OC.

**Figure 3:**
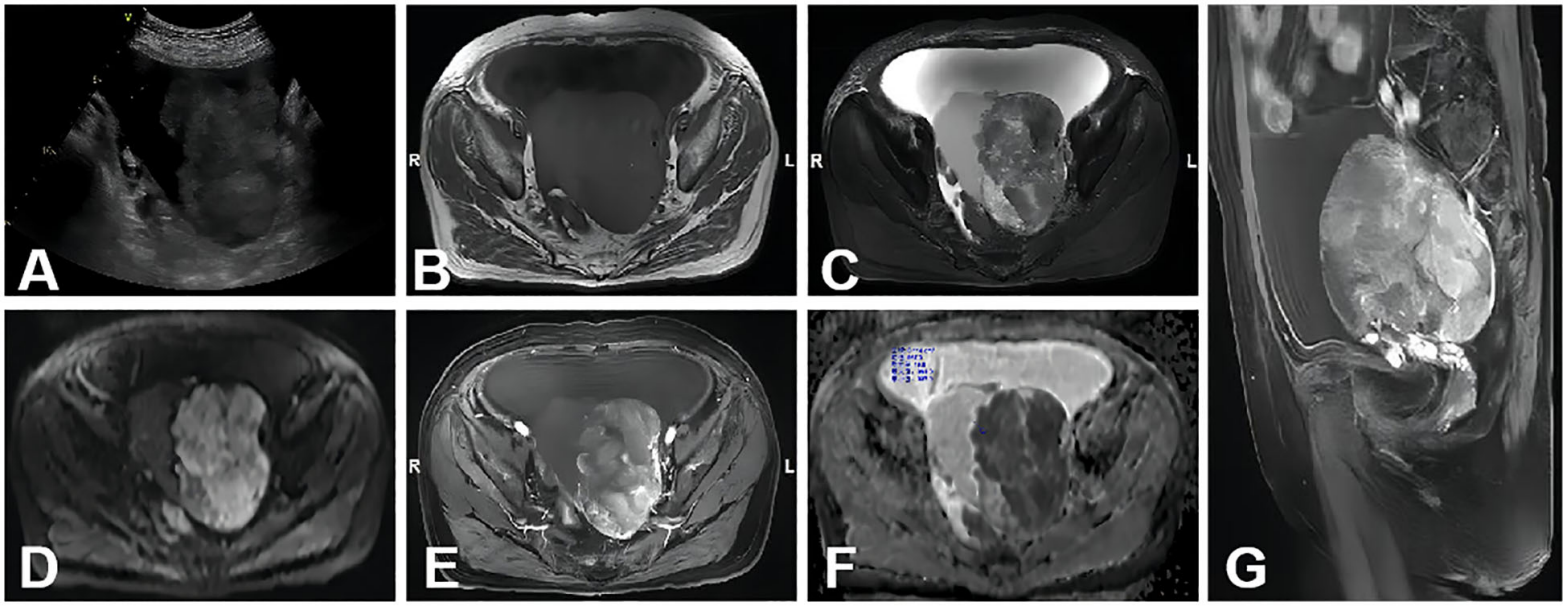
A 70-year-old woman with stage I CCC on the left ovary. **(A)** A non-homogeneous middle-hypoechoic solid mass was detected by ultrasound examination. **(B–D)** A lobulated cystic-solid mass with hemorrhage signal. The large solid portion showed mainly isointense, hyperintense, and hyperintense signal intensities on axial T1WI, T2WI, and DWI (b=1,000 s/mm^2^), respectively. **(E)** Axial contrast-enhanced T1WI showed that the solid portion was of intensive enhancement. **(F)** On the ADC map, the mean ADC value was 0.719 × 10^−3^ mm^2^/s. **(G)** Sagittal contrast enhanced T1WI showed the mass was similar to **(E)**.

Compared with other types of malignant ovarian tumors, CCC exhibited a higher ADC value, consistent with findings in previous studies (29, 30) (**Figure 3**). Additionally, the typical features of ovarian CCC and EC were hemorrhage signals found in these masses. It may be due to the abovementioned two types of ovarian cancer are highly associated with endometriosis (31, 32). Radiologists can observe the characteristic hemorrhage signal inside the mass on MRI and assist in diagnosis. According to another study (33), the utilization of morphological characteristics observed on MRI, such as a round mural nodule exhibiting a high “Height-to-Width ratio” and a focal growth pattern, proves to be valuable in differentiating CCC from EC.

Besides the abovementioned types of ovarian tumors, this group also included four cases HGSOC and one YST. MRI provides a more comprehensive evaluation of organs in pelvis and improve the diagnostic accuracy of HGSOC. In the case of the 26-year-old patient diagnosed with stage I YST, characterized by markedly elevated AFP levels, the ovarian mass exhibited significant enhancement along with multiple signal voids, indicative of its hypervascular nature. These findings are consistent with those reported in prior studies (34, 35).

### The IOTA-ADNEX model’s challenge in discriminating between primary and metastatic tumors

**Figure 4:**
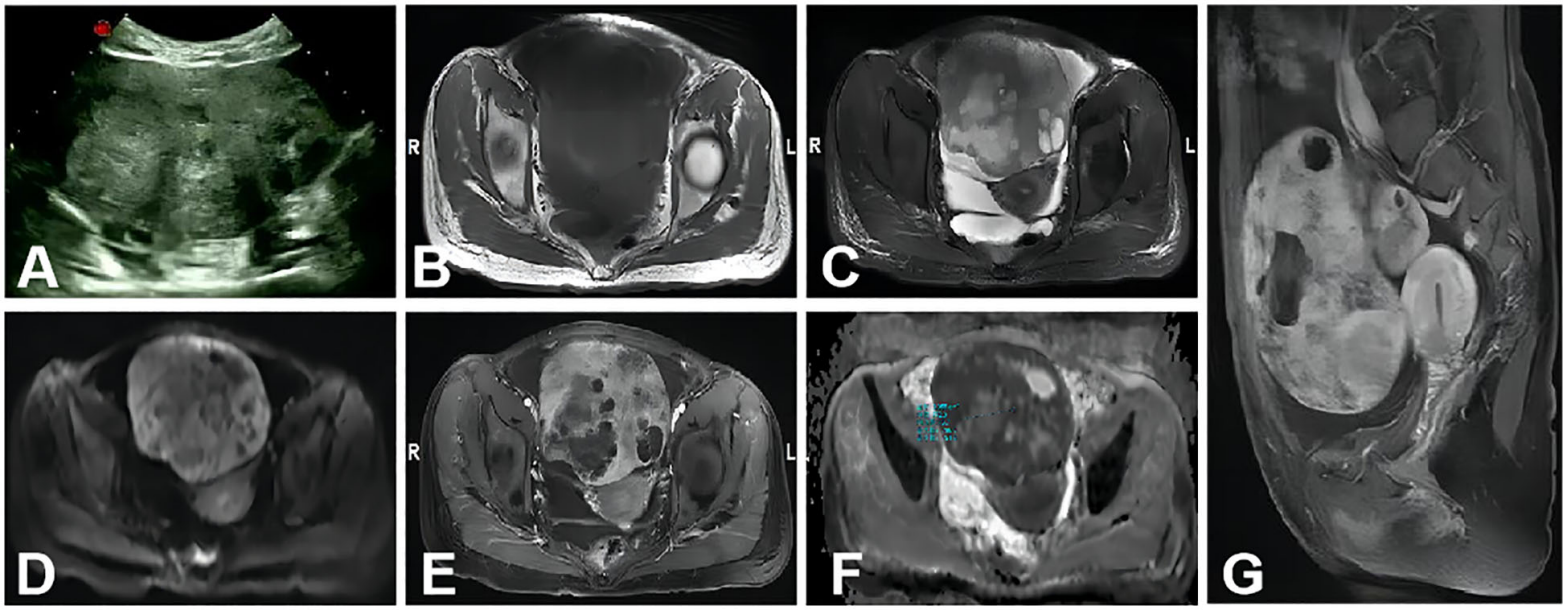
A 34-year-old woman with metastasis of gastric cancer to the bilateral ovaries. The large mass in the abdominal-pelvic cavity was from the right ovary. **(A)** A non-homogeneous middle-hypoechoic solid mass was detected by ultrasound examination. **(B–D)** A solid mass with clear border and necrosis shows mainly isointense, hyperintense, and hyperintense signal intensities on axial T1WI, T2WI, and DWI (b=1,000s/mm^2^), respectively. **(E)** Axial contrast-enhanced T1WI showed the solid portion was intensive enhancement. **(F)** On the ADC map, the mean ADC value was 0.772 × 10^−3^ mm^2^/s. **(G)** Sagittal contrast-enhanced T1WI showed that the mass was similar to **(E)**.

Preoperative differentiation between ovarian cancer, particularly stages II–IV, and secondary cancers of the adnexa remains a challenge, even with the use of the ADNEX model. Research conducted in oncology centers in China and Brazil (14, 15) demonstrated that the AUC for distinguishing stage I ovarian OC from metastasis was 0.81 and 0.64, respectively, whereas the AUC for stage II–IV OC versus metastasis was 0.78 and 0.89. In this study, seven instances of metastatic ovarian cancer were incorrectly classified as primary ovarian cancer by the ADNEX model. Further analysis uncovered three cases of gastric cancer metastasis, two colorectal, one endometrial, and one cervical cancer metastasis among the misclassified cases. The imaging of the gastric cancer metastases revealed solid masses with intense enhancement, necrotic regions, and clear separation from adjacent structures (**Figure 4**). MRI can provide a superior assessment of the neighboring organs for the latter three tumor types, encompassing the uterine cervix, endometrium, and sigmoid colon. Additionally, the study reported a misclassification of a stage I dysgerminoma as metastatic by the ADNEX model. This mass revealed characteristic fibrovascular septa on MRI, findings that are in agreement with those reported in the study (36).

A previous study (37) has indicated that patients with metastatic ovarian tumors tend to be younger and present with lower levels of CA125 and HE-4 compared with those with primary ovarian tumors. However, in the current study, a significant difference in CEA levels was observed among the 51 patients who were mis-subclassified by the ADNEX model. The absence of significant distinctions in age and CA125, both variables integrated into the model, could potentially account for the model’s inaccuracies in sub-classification.

### Strengths and weaknesses of MRI for the diagnosis of ovarian masses

When ultrasound imaging fails to qualitatively diagnose an ovarian mass, MRI offers the advantage of providing further assessment for “indeterminate adnexal masses at ultrasound”. The MRI characteristics of the mass contribute to the identification of specific ovarian tumor types and offer a comprehensive assessment of ovarian masses and adjacent organ involvement. However, MRI scanning is relatively slow, incurs high costs, and requires considerable time for scheduling. Additionally, MRI tends to produce motion artifacts and is not superior to contrast-enhanced computed tomography in detecting peritoneal metastasis and ascites (38).

There are several limitations in the study. Firstly, the lack of a control study design indicates a need for further research with a prospective design. Secondly, the small sample size could have impacted the results.

In conclusion, our study showed that DWI signals, ADC values, enhancement levels of the solid portion, and characteristic components within the mass on MRI images can provide more supplementary information for malignant ovarian tumors mis-classified by the ADNEX model. We hope this effort will contribute to enhancing the preoperative diagnostic accuracy for patients with malignant ovarian tumors.

## Data Availability

The raw data supporting the conclusions of this article will be made available by the authors, without undue reservation.
