# Supplementary material for: Diagnostic value of magnetic resonance imaging for malignant ovarian tumors mis-subclassified by the ultrasound-based ADNEX model
Source: Front Oncol. 2025 Feb 25;15:1406735. doi: 10.3389/fonc.2025.1406735 (PMC11893382; doi:10.3389/fonc.2025.1406735)
Supplement: Supplementary file 1 [file Table1.docx]

Supplementary Table 1 MRI findings of 51 malignant adnexal masses mis-subclassified by the IOTA-ADNEX model

| MRI characteristics | | Primary ovarian tumors | | | | | | | | Metastatic ovarian tumors | | | | | |
| --- | --- | --- | --- | --- | --- | --- | --- | --- | --- | --- | --- | --- | --- | --- | --- |
|  |  | HGSOC(n=5) | CCC  (n=14) | EC  (n=2) | MC  (n=4) | GCT  (n=2) | Yolk sac tumor  (n=1) | Immature  teratoma  (n=1) | Dysgerminoma  (n=1) | colorectal cancer (n=10) | Gastric cancer (n=4) | Uterine cervical cancer(n=2) | Breast cancer (n=1) | Endometrial cancer  (n=3) | LAMN  (n=1) |
| Size (cm) | | 7.55±  3.252 | 10.90±5.826 | 11.24±1.499 | 12.72±4.295 | 5.79±  3.083 | 4.74 | 8.70 | 20.06 | 8.71±  5.264 | 8.44±  3.075 | 7.10±  4.808 | 8.37 | 6.82±  5.822 | 16.29 |
| shape | lobulated | 5(1.00) | 11(0.79) | 2(1.00) | 3(0.75) | 1(0.50) | 1(1.00) | 0(0.00) | 1(1.00) | 9(0.90) | 2(0.50) | 2(1.00) | 1(1.00) | 3(1.00) | 1(1.00) |
|  | round | 0(0.00) | 3(0.21) | 0(0.00) | 1(0.25) | 1(0.50) | 0(0.00) | 1(1.00) | 0(0.00) | 1(0.10) | 2(0.50) | 0(0.00) | 0(0.00) | 0(0.00) | 0(0.00) |
| Composition of the mass | solid | 3(0.60) | 0(0.00) | 0(0.00) | 0(0.00) | 1(0.50) | 1(1.00) | 0(0.00) | 1(1.00) | 5(0.50) | 4(1.00) | 1(0.50) | 0(0.00) | 2(0.67) | 0(0.00) |
|  | Solidcystic | 2(0.40) | 14(1.00) | 2(1.00) | 2(0.50) | 0(0.00) | 0(0.00) | 1(1.00) | 0(0.00) | 3(0.30) | 0(0.00) | 0(0.00) | 1(1.00) | 1(0.33) | 0(0.00) |
|  | Multilocularcystic | 0(0.00) | 0(0.00) | 0(0.00) | 2(0.50) | 1(0.50) | 0(0.00) | 0(0.00) | 0(0.00) | 2(0.50) | 0(0.00) | 1(0.50) | 0(0.00) | 0(0.00) | 1(1.00) |
| T1-signal | Hyper- | 0(0.00) | 0(0.00) | 0(0.00) | 1(0.25) | 0(0.00) | 0(0.00) | 0(0.00) | 0(0.00) | 0(0.00) | 0(0.00) | 1(0.50) | 0(0.00) | 0(0.00) | 0(0.00) |
|  | Iso- | 5(1.00) | 14(1.00) | 2(1.00) | 3(0.75) | 2(1.00) | 1(1.00) | 1(1.00) | 1(1.00) | 10(1.00) | 4(1.00) | 1(0.50) | 1(1.00) | 3(1.00) | 1(1.00) |
|  | Hypo- | 0(0.00) | 0(0.00) | 0(0.00) | 0(0.00) | 0(0.00) | 0(0.00) | 0(0.00) | 0(0.00) | 0(0.00) | 0(0.00) | 0(0.00) | 0(0.00) | 0(0.00) | 0(0.00) |
| T2-signal | Hyper- | 5(1.00) | 14(1.00) | 2(1.00) | 4(1.00) | 2(1.00) | 1(1.00) | 1(1.00) | 1(1.00) | 9(0.90) | 4(1.00) | 2(1.00) | 1(1.00) | 3(1.00) | 1(1.00) |
|  | Iso- | 0(0.00) | 0(0.00) | 0(0.00) | 0(0.00) | 0(0.00) | 0(0.00) | 0(0.00) | 0(0.00) | 1(0.10) | 0(0.00) | 0(0.00) | 0(0.00) | 0(0.00) | 0(0.00) |
|  | Hypo- | 0(0.00) | 0(0.00) | 0(0.00) | 0(0.00) | 0(0.00) | 0(0.00) | 0(0.00) | 0(0.00) | 0(0.00) | 0(0.00) | 0(0.00) | 0(0.00) | 0(0.00) | 0(0.00) |
| DWI-signal | Hyper- | 5(1.00) | 14(1.00) | 2(1.00) | 4(1.00) | 2(1.00) | 1(1.00) | 1(1.00) | 1(1.00) | 10(1.00) | 4(1.00) | 2(1.00) | 1(1.00) | 3(1.00) | 1(1.00) |
|  | Iso- | 0(0.00) | 0(0.00) | 0(0.00) | 0(0.00) | 0(0.00) | 0(0.00) | 0(0.00) | 0(0.00) | 0(0.00) | 0(0.00) | 0(0.00) | 0(0.00) | 0(0.00) | 0(0.00) |
|  | Hypo- | 0(0.00) | 0(0.00) | 0(0.00) | 0(0.00) | 0(0.00) | 0(0.00) | 0(0.00) | 0(0.00) | 0(0.00) | 0(0.00) | 0(0.00) | 0(0.00) | 0(0.00) | 0(0.00) |
| ADC value (×10^−3^ mm^2^ /s) | | 0.63±  0.092 | 1.01±  0.132 | 0.67±  0.053 | 0.77±  0.069 | 0.74±  0.201 | 0.78 | 0.75 | 0.81 | 0.79±  0.147 | 0.81±  0.179 | 0.84±  0.105 | 0.64 | 0.67±  0.099 | / |
| Enhancement | slight | 0(0.00) | 0(0.00) | 1(0.50) | 0(0.00) | 0(0.00) | 0(0.00) | 0(0.00) | 0(0.00) | 0(0.00) | 0(0.00) | 1(0.50) | 0(0.00) | 0(0.00) | 0(0.00) |
|  | moderate | 2(0.40) | 2(0.14) | 1(0.50) | 2(0.50) | 1(0.50) | 0(0.00) | 1(1.00) | 1(1.00) | 3(0.30) | 0(0.00) | 1(0.50) | 0(0.00) | 2(0.67) | 0(0.00) |
|  | intensive | 3(0.60) | 12(0.86) | 0(0.00) | 2(0.50) | 1(0.50) | 1(1.00) | 0(0.00) | 0(0.00) | 7(0.70) | 4(1.00) | 0(0.00) | 1(1.00) | 1(0.33) | 1(1.00) |
| Necrosis | yes | 3(0.60) | 1(0.07) | 0(0.00) | 1(0.25) | 0(0.00) | 0(0.00) | 0(0.00) | 1(1.00) | 5(0.50) | 4(1.00) | 1(0.50) | 1(1.00) | 0(0.00) | 0(0.00) |
|  | no | 2(0.40) | 13(0.93) | 2(1.00) | 3(0.75) | 2(1.00) | 1(1.00) | 1(1.00) | 0(0.00) | 5(0.50) | 0(0.00) | 1(0.50) | 0(0.00) | 3(1.00) | 1(1.00) |
| Hemorrhage | yes | 1(0.20) | 10(0.71) | 2(1.00) | 0(0.00) | 2(1.00) | 0(0.00) | 0(0.00) | 0(0.00) | 2(0.20) | 0(0.00) | 0(0.00) | 1(1.00) | 1(0.33) | 0(0.00) |
|  | no | 4(0.80) | 4(0.29) | 0(0.00) | 4(1.00) | 0(0.00) | 1(1.00) | 1(1.00) | 1(1.00) | 8(0.80) | 4(1.00) | 2(1.00) | 0(0.00) | 2(0.67) | 1(1.00) |
| O-RADS score | 3 (n=1) | 0(0.00) | 1(0.07) | 0(0.00) | 0(0.00) | 0(0.00) | 0(0.00) | 0(0.00) | 0(0.00) | 0(0.00) | 0(0.00) | 0(0.00) | 0(0.00) | 0(0.00) | 0(0.00) |
|  | 4 (n=15) | 2(0.04) | 1(0.07) | 2(1.00) | 2(0.50) | 1(0.50) | 0(0.00) | 1(1.00) | 1(1.00) | 3(0.30) | 0(0.00) | 0(0.00) | 0(0.00) | 2(0.67) | 0(0.00) |
|  | 5 (n=35) | 3(0.06) | 12(0.86) | 0(0.00) | 2(0.50) | 1(0.50) | 1(1.00) | 0(0.00) | 0(0.00) | 7(0.70) | 4(1.00) | 2(1.00) | 1(1.00) | 1(0.33) | 1(1.00) |

HGSOC: high-grade serous ovarian carcinoma; CCC: clear cell carcinoma; EC: endometrioid carcinoma; MC: mucinous carcinoma; GCT: granulosa cell tumor YST: yolk sac tumor; LAMN: low-grade appendiceal mucinous neoplasm; MRI: magnetic resonance imaging; DWI: diffusion-weighted Imaging; ADC: apparent diffusion coefficient; O-RADS: Ovarian-Adnexal Reporting and Data System.

*The solid component of the mass is too small, resulting in a failed ADC measurement.
